# Mapping prostatic microscopic anisotropy using linear and spherical b-tensor encoding: A preliminary study

**DOI:** 10.1002/mrm.28856

**Published:** 2021-05-31

**Authors:** Markus Nilsson, Greta Eklund, Filip Szczepankiewicz, Mikael Skorpil, Karin Bryskhe, Carl-Fredrik Westin, Claes Lindh, Lennart Blomqvist, Fredrik Jäderling

**Affiliations:** 1 Clinical Sciences Lund, Radiology, Lund University, Lund, Sweden; 2 Random Walk Imaging AB, Lund, Sweden; 3 Clinical Sciences Lund, Medical Radiation Physics, Lund University, Lund, Sweden; 4 Department of Molecular Medicine and Surgery, Karolinska Institutet, Stockholm, Sweden; 5 Department of Neuroradiology, Karolinska University Hospital, Solna, Stockholm, Sweden; 6 Brigham and Women’s Hospital, Harvard Medical School, Boston, Massachusetts, USA; 7 Department of Oncology-Pathology, Karolinska Institutet, Stockholm, Sweden; 8 Department of Diagnostic Radiology, Karolinska University Hospital, Solna, Sweden; 9 Department of Radiology, Capio S:t Görans Hospital, Stockholm, Sweden

**Keywords:** diffusion-weighted imaging, heterogeneity, microscopic anisotropy, prostate cancer, tensor-valued diffusion encoding

## Abstract

**Purpose:**

Tensor-valued diffusion encoding provides more specific information than conventional diffusion-weighted imaging (DWI), but has mainly been applied in neuroimaging studies. This study aimed to assess its potential for the imaging of prostate cancer (PCa).

**Methods:**

Seventeen patients with histologically proven PCa were enrolled. DWI of the prostate was performed with linear and spherical tensor encoding using a maximal b-value of 1.5 ms/μm^2^ and a voxel size of 3 × 3 × 4 mm^3^. The gamma-distribution model was used to estimate the mean diffusivity (MD), the isotropic kurtosis (MK_I_), and the anisotropic kurtosis (MK_A_). Regions of interest were placed in MR-defined cancerous tissues, as well as in apparently healthy tissues in the peripheral and transitional zones (PZs and TZs).

**Results:**

DWI with linear and spherical encoding yielded different image contrasts at high b-values, which enabled the estimation of MK_A_ and MK_I_. Compared with healthy tissue (PZs and TZs combined) the cancers displayed a significantly lower MD (*P* < .05), higher MK_I_ (*P* < 10^−5^), and lower MK_A_ (*P* < .05). Compared with the TZ, tissue in the PZ showed lower MD (*P* < 10^−3^) and higher MK_A_ (*P* < 10^−3^). No significant differences were found between cancers of different Gleason scores, possibly because of the limited sample size.

**Conclusion:**

Tensor-valued diffusion encoding enabled mapping of MK_A_ and MK_I_ in the prostate. The elevated MK_I_ in PCa compared with normal tissues suggests an elevated heterogeneity in the cancers. Increased in-plane resolution could improve tumor delineation in future studies.

## 1 | INTRODUCTION

Prostate cancer (PCa) is the second most frequent cancer diagnosis in men, and the fifth leading cause of death world-wide.^1^ In the Nordic countries, the lifetime risk for a man to be diagnosed with PCa is approximately 1 in 6, and the risk of dying from the disease is 1 in 30.^2^ There is a large variation in cancer aggressiveness and prognosis, which poses a challenge for diagnosis and treatment planning. The introduction of MRI in combination with targeted biopsies in the diagnostic workflow has been a paradigm shift,^3,4^ leading to a reduction of the number of performed biopsies. Compared with systematic biopsies alone, the use of MRI and targeted biopsies increase detection of significant cancers and reduce the overdetection of indolent low-grade cancers.^5,6^

A cornerstone for the detection of significant cancers with MRI is diffusion-weighted imaging (DWI). Apart from providing images for radiological assessment, it also provides maps of potential quantitative imaging biomarkers. A simple but powerful example is the apparent diffusion coefficient (ADC), which correlates with cellularity in many different types of cancers.^7^ Another is the fractional anisotropy (FA) obtained from diffusion tensor imaging (DTI).^8^ This parameter is sensitive to the presence of elongated cell structures and has been proposed to improve the detection and grading of PCa.^9,10^ However, previous studies found contradicting results, with FA being either higher or lower in PCas compared with normal glandular tissue.^10–12^ This variation could be caused by multiple factors. One is that prostatic FA tends to decrease with echo time and increase with diffusion time,^13,14^ and these imaging parameters can differ between studies. Another factor is the limited spatial resolution of MRI, which causes each imaging voxel to include cell structures with different orientations. This in itself reduces the FA^15,16^ and leads to a voxel-size dependence of the FA in organs with high orientation dispersion such as the prostate.^17^ To resolve this problem, anisotropy must be detected on the micrometer level, rather than on the millimeter level as is done in DTI.

Tensor-valued diffusion encoding has recently been proposed as a viable approach for the estimation of microscopic diffusion anisotropy.^18–20^ The approach is based on two pillars. First, it uses high b-value DWI to reveal the effects of non-Gaussian diffusion,^21^ which in this context refers to the presence of multiple components undergoing approximately Gaussian diffusion. Second, it uses two different shapes of the diffusion encoding tensor to separate two contributions to the multi-Gaussian diffusion: microscopic diffusion anisotropy and isotropic heterogeneity. These contributions are quantified in terms of the anisotropic kurtosis (MK_A_) and isotropic kurtosis (MK_I_), respectively.^16,19^ In brain tumors, these parameters have a link to features from histology slides^22^: between MK_A_ and the density of elongated cell structures and between MK_I_ and the intravoxel variation in cell density. Tensor-valued diffusion encoding has been applied to study microscopic anisotropy in a wide range of conditions, including schizophrenia,^19^ brain tumors,^22,23^ epilepsy,^24^ and kidney variations.^25^ It has also been used to learn about cerebellar structure^26^ and in conjunction with motion-compensated encoding to improve DWI of the heart.^27,28^ However, tensor-valued encoding has so far not been applied in the context of PCa.

The purpose of this study was to test the potential of tensor-valued diffusion encoding for imaging of the prostate in patients with PCa, and to provide an initial survey of the microscopic diffusion anisotropy and isotropic heterogeneity in this organ and its tumors. Our hypothesis was that tissues of the prostate with more elongated cell structures would exhibit higher microscopic diffusion anisotropy (MK_A_), and that cancers that often exhibit a relatively chaotic microenvironment would exhibit higher isotropic heterogeneity (MK_I_) than normal-appearing tissue.

## 2 | METHODS

### 2.1 | Patient inclusion and preparation

Seventeen patients with histologically proven PCas were included in the study, after providing written informed consent to participation. The study was approved by the regional ethical review board in Stockholm (Dnr. 2013/1655-31/2). Patients underwent a prostate MRI before surgery on a Siemens 3T scanner (Siemens Magnetom, Skyra). Patients were advised to refrain from sexual activity with ejaculation 3 days before the examination to increase fluid content in the peripheral zone (PZ) and seminal vesicles. On the day of the examination, patients were instructed to fasten and refrain from nicotine-containing substances 1 h before examination to reduce bowel movements. All patients had a bowel preparation 2 h before examination using a small enema Microlax (McNeil Sweden AB). In some patients, a small catheter was introduced to evacuate gas in the rectum to minimize image artifacts. One milligram of Glucagon (Novo Nordisk Scandinavia AB) was administered intramuscularly just before the examination to reduce bowel motility. The body coil was placed over the small pelvis (Body 18; Siemens) and run together with a spine coil (Spine Coil 32; Siemens). After MRI, all patients underwent robot-assisted laparoscopic radical prostatectomy using a three-armed DaVinci system (Intuitive Surgical).

### 2.2 | MRI and statistical analysis

**FIGURE 1:**
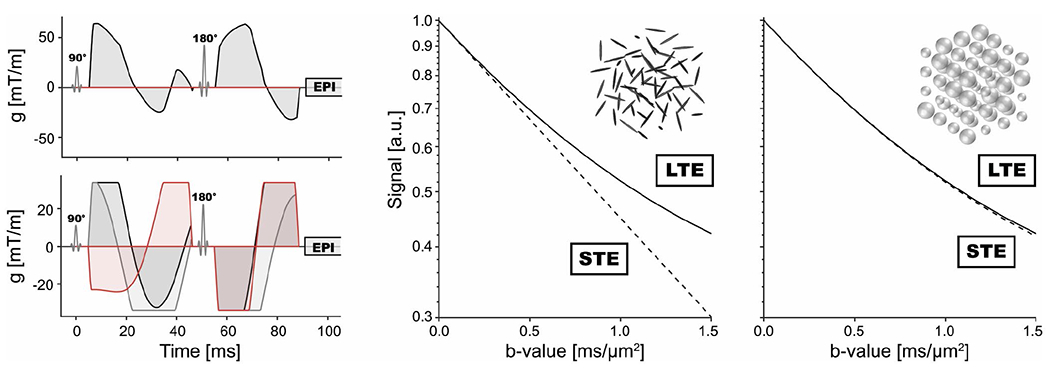
Method overview. The leftmost panels show gradient waveforms used for linear tensor encoding (LTE) and spherical tensor encoding (STE) at the top and bottom, respectively. For LTE, waveforms are applied in one direction only, whereas STE waveforms are applied in all three spatial dimensions (represented by red, dark-gray, and light-gray waveforms, respectively). In addition to the diffusion-encoding waveforms, the plots also show the radiofrequency pulses (90° and 180°) and the imaging readout block (echo-planar imaging). The middle and right panels show diffusion-weighted signal curves for a synthetic voxel comprised of environments in which the diffusion is described by anisotropic but randomly ordered diffusion tensors (middle) and isotropic diffusion tensors with variable diffusivity (right). Note that the diffusion is isotropic on the voxel level in both cases. For the case with microscopically anisotropic diffusion, the curves from LTE and STE deviate at higher b-values, whereas they overlap for the other case. This provides a mechanism to disentangle microscopic anisotropy (MK_A_) from isotropic heterogeneity (MK_I_)

MRI was performed using a T_2_-weighted turbo-spin–echo sequence (voxel size = 0.5 × 0.5 mm^2^, slice thickness = 3 mm, TR = 4590 ms, TE = 109 ms), and DWI (voxel size = 1.5 × 1.5 mm^2^, slice thickness = 4.8 mm, TR = 6100 ms, TE = 63 ms). In addition, DWI was performed using a prototype spin-echo echo-planar imaging sequence, customized for tensor-valued diffusion encoding with user-defined free gradient waveforms.^27^ Data were acquired with two different types of gradient waveforms. These were optimized to yield linear (LTE) and spherical (STE) diffusion-encoding tensors.^29^ Each waveform was used to acquire five distinct b-values (0.2, 0.5, 0.8, 1.2, and 1.5 ms/μm^2^) in eight different directions. Figure 1 shows the waveforms used and how they support estimation of microscopic anisotropy and isotropic heterogeneity. In total, each data set comprised 80 image volumes acquired in approximately 6 min. All images were measured using a matrix size = 128 × 128, 17 slices, in-plane voxel size = 3 × 3 mm^2^, slice thickness = 4 mm, TR = 4000 ms, and TE = 101 ms. Image registration was applied to correct for motion and eddy current artifacts, using ElastiX.^30^ To avoid errors introduced by the registration algorithm itself, only in-plane adjustments were allowed.

The data acquired with tensor-valued diffusion encoding were analyzed to yield the two different types of diffusional kurtosis supported by this acquisition.^19,31^ Prior to analysis, data were averaged across the diffusion encoding directions (referred to as powder averaging).^31,32^ The following equation was then fitted to the data^31^:

$$\text{S}/{\text{S}}_{0}={\left(1+\frac{1}{3}\text{b}\cdot \mathrm{MD}\cdot \mathrm{MK}\right)}^{-3/\mathrm{MK}}$$

where S_0_ is the nondiffusion-weighted signal intensity, MD is the mean diffusivity, $\text{MK}={\mathrm{MK}}_{\text{I}}+{\text{b}}_{\text{Δ}}^{2}{\mathrm{MK}}_{\text{A}}$, and MK_A_ is the anisotropic kurtosis, MK_I_ is the isotropic kurtosis, and b_Δ_ is the shape of the b-tensor, which is zero for STE and unity for LTE. The open-source analysis software is available at https://github.com/markus-nilsson/md-dmri,^33^ (SHA-1 hash 989ebab), with this method referred to as dtd_gamma.

Regions of interest (ROIs) were manually drawn on the high-resolution DWI and T2-weighted (T_2_W) images by an experienced radiologist (FJ), after which they were transferred to the low-resolution space of the images acquired with LTE and STE, corresponding to tumor locations on histology slides. The transform was obtained from a rigid body registration from the high- to the low-resolution space using ElastiX. Three types of ROIs were defined. The first covered the tumor lesions (one per lesion, up to three different ROIs per patient). In total, 32 histologically proven PCas were defined (7 in the transitional zone (TZ) and 25 in the PZ). The second and third covered normal-appearing regions with no tumor at the corresponding location at histology of the TZs and PZs. From each ROI, values were extracted of the three diffusion parameters (MD, MK_I_, MK_A_). The values were then assigned to one of five groups: cancers with Gleason score of 3 + 3, 3 + 4, or 4 + 3, and TZ or PZ. Three types of comparisons were performed: between each cancer type, between cancer and healthy-appearing prostate tissue (3 + 3, 3 + 4, and 4 + 3 vs TZ and PZ), and between TZ and PZ. Tests for differences in medians were done using nonparametric Wilcoxson rank-sum tests (chosen because of limited sample sizes), with a significance threshold of 0.05.

### 2.3 | Postoperative pathology

All prostatic tissue was fixed in 10% buffered formaldehyde. The specimens were subjected to the whole-mount, step-section according to the Stanford procedure and sliced horizontally, perpendicular to the dorsal aspect of the prostate in 3- to 4-mm slices. The slicing corresponded to the angle of the transversal images on MRI, augmenting the correlation of tumor location at the base, mid, and apex. The slices were subsequently stained with hematoxylin-eosin for tissue microstructure visualization.

## 3 | RESULTS

**FIGURE 2:**
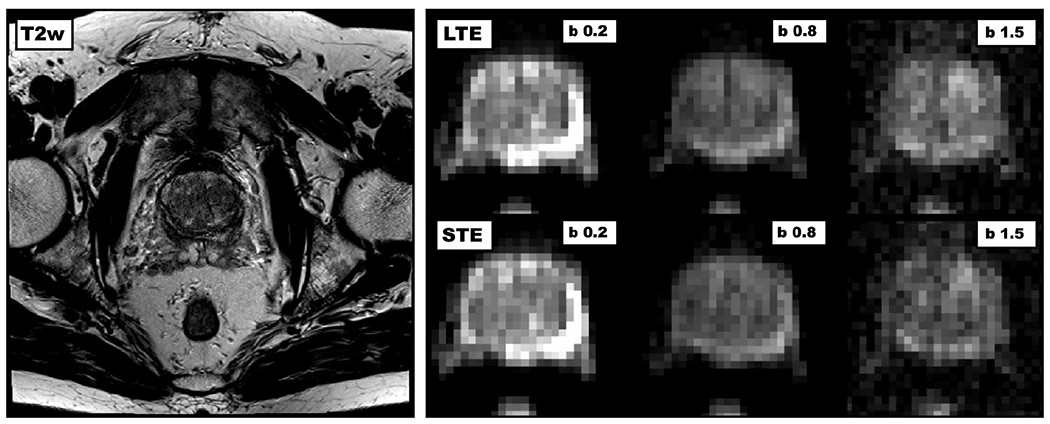
Overview of acquired images. The left image shows a T_2_-weighted transversal slice across the pelvis from the turbo-spin–echo sequence. The right image shows cropped versions of the diffusion-weighted images, where the columns show different b-values and the rows different shapes of the b-tensor. The contrast obtained with linear tensor encoding (top row) and spherical tensor encoding (bottom row) is similar at low b-values, but starts to deviate at higher b-values. The b-values are reported in units of ms/μm^2^. LTE, linear tensor encoding; STE, spherical tensor encoding; T2w, T_2_-weighted

**FIGURE 3:**
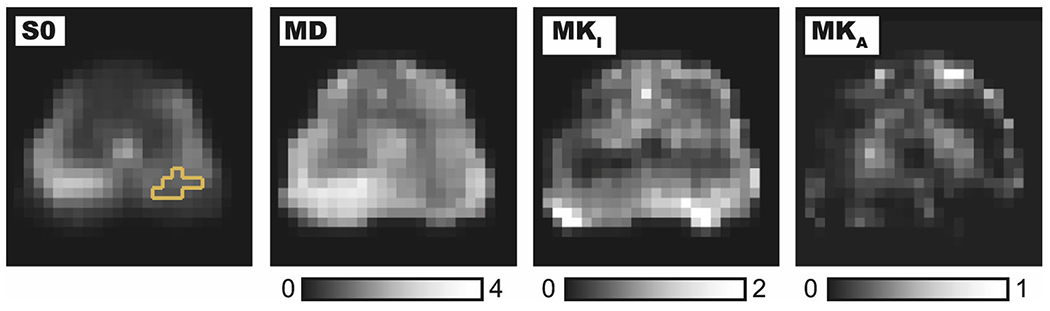
Overview of parameter maps. Columns show fitted T_2_-weighted signal intensity (S0) with the tumor region of interest marked in yellow, mean diffusivity (MD in units of μm^2^/ms), isotropic kurtosis (MK_I_), and anisotropic kurtosis (MK_A_). High MK_A_ indicating high microscopic diffusion anisotropy is found symmetrically around the midline. The high values of MK_A_ in the top part of the image are probably caused by a low signal-to-noise ratio because it coincides with the low values of S_0_

A T_2_-weighted image and a set of DWIs acquired at different b-values with LTE and STE are shown in Figure 2. The appearance of the LTE and STE images is similar at low b-values, as expected, whereas subtle contrast differences appear at higher b-values. The LTE and STE data were jointly analyzed to obtain the parameter maps that are shown in Figure 3. All parameters are unitless, except for MD, which is given in units of μm^2^/ms. Three of the maps (*S*_0_, MD, and MK_I_) are most intense in the PZ, whereas one (MK_A_) is most intense in the TZ and in the anterior fibromuscular stroma.

**FIGURE 4:**
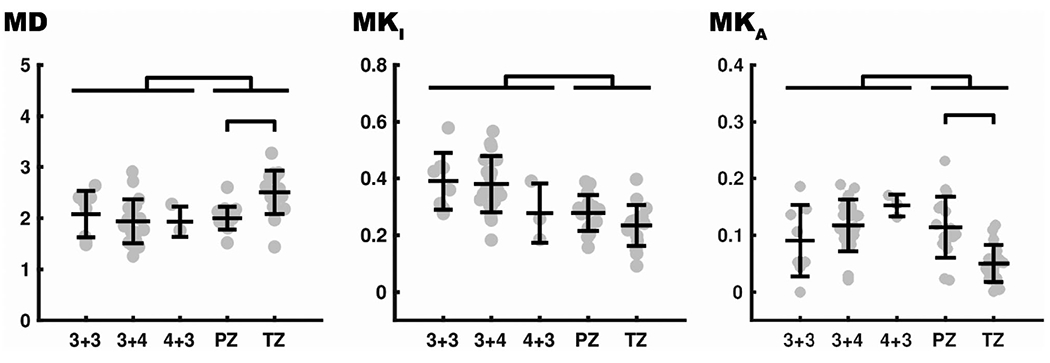
Quantitative analysis of prostatic tumors versus healthy tissue. A statistically significant difference was found between the cancers (with Gleason scores of 3 + 3, 3 + 4, and 4 + 3) and healthy tissue (peripheral zone [PZ] and transitional zone [TZ]) for mean diffusivity (MD; *P* < .05), isotropic kurtosis (MK_I_; *P* < 10^−5^), and anisotropic kurtosis (MK_A_; *P* < .05). Significant differences were found between PZ and TZ for MD (*P* < 10^−3^) and for MK_A_ (*P* < 10^−3^). No significant differences were found between cancers with different Gleason scores

Values of MD, MK_I_, and MK_A_ for prostate cancer tissue (characterized by the Gleason score) and in healthy prostate tissue within both the PZ and the TZ are shown in Figure 4. The distributions of MK_I_ and MK_A_ are summarized by their mean and variance. Cancer tissues showed a significantly lower MD (*P* < .05), higher MK_I_ (*P* < 10^−5^), and higher MK_A_ (*P* < .05) compared with normal tissue (TZ and PZ combined). Moreover, compared with healthy tissue in the TZ, we found that the PZ showed lower MD (*P* < 10^−3^) and higher MK_A_ (*P* < 10^−3^). No significant differences were found between tumors of different Gleason scores.

**FIGURE 5:**
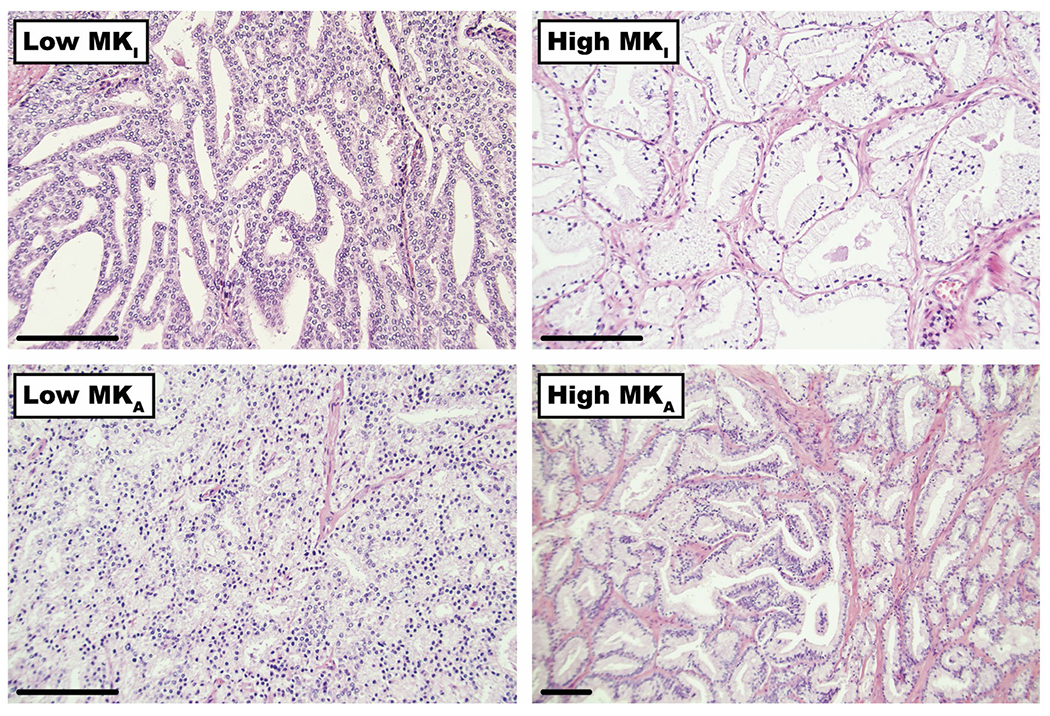
Examples of histology slices. The slices were obtained from selected cases with lowest and highest values of isotropic kurtosis (MK_I_) and anisotropic kurtosis (MK_A_). Top row shows examples of cancers having low MK_I_ to the left (Gleason score = 4 + 4, peripheral zone [PZ]) and high MK_I_ (Gleason score = 3 + 4, transitional zone [TZ]) to the right. The higher MK_I_ seems to be related to a higher amount of stroma surrounding the ducts, which appears to add to the heterogeneity in diffusion coefficients. Bottom row shows examples of cancers having low MK_A_ to the left (Gleason score = 4 + 4, TZ) and high MK_A_ to the right (Gleason score = 3 + 4, TZ). High MK_A_ appears to have a higher prevalence of elongated cellular structures. Scale bars = 100 μm

Figure 5 shows hematoxylin-eosin–stained histopathological data from cancers with values of MK_I_ and MK_A_ in the lowest and highest percentiles of all cases. The observed stroma surrounding the ducts in the high MK_I_ case appears to add to the heterogeneity in diffusion coefficients compared with the low MK_I_ case where the tissue microstructure is more uniform. In the high MK_A_ case, the tissue appears to have a higher prevalence of elongated cellular structures compared with the case with low MK_A_.

## 4 | DISCUSSION

This study aimed to test the feasibility of tensor-valued diffusion encoding in the prostate to map tissue heterogeneity and microscopic anisotropy by the isotropic and anisotropic kurtosis (MK_I_ and MK_A_). Results showed plausible maps (Figure 3), where the PZ showed high values of MK_I_, indicating a heterogeneous microenvironment with compartments having a wide variation of diffusivities within the voxel.^22^ On average, the PZ was found to have significantly higher MK_A_ than the TZ (Figure 3), but as we can see in Figure 3 the transitional zone showed a mix of high and low values of MK_A_. Higher values of MK_A_ are expected where there is a larger presence of elongated cell structures.^22^ In the prostate, regions with stromal smooth muscle have high diffusion anisotropy on the microscopic level.^17^ However, this only leads to high values of the FA obtained with DTI if the resolution is high enough—otherwise, the high dispersion in orientation between muscle fibers in the voxel leads to low values of FA.^17^ Using tensor-valued encoding, we can detect and quantify the level of microscopic diffusion anisotropy even in the presence of orientation dispersion.^16,31^ Thus, we interpret regions of the TZ with high MK_A_ as regions with high amounts of smooth muscle. In the cancers, histology shows how discrete well-formed glands are replaced with poorly formed and fused glands as the cancers progress from Gleason pattern 3 to pattern 4.^34^ This can be seen as a progression towards a more disorganized and heterogeneous tissue, which is aligned with the finding of increased MK_I_ in the cancers. However, predicting MRI parameters based on histology is no easy task because tissue components contribute not by their volume fractions alone but rather by their T_2_-weighted signal fractions.^13^ Future studies could combine tensor-valued encoding with acquisitions featuring multiple echo times to further increase specificity,^35^ which could be particularly useful because diffusion-relaxation correlation experiments can improve prostate characterization.^36,37^

The question of diagnostic relevance is whether the cancers exhibited characteristics that were different from those of the healthy tissue. Indeed, we found lower MD, higher MK_I_ and higher MK_A_ in the cancers, with the strongest effect found for MK_I_ (Figure 4). Some of the cancers had values of MK_I_ that were considerably higher than any of the values in PZ and TZ. Future studies with larger sample sizes could assess the receiver-operating characteristic curve for using MK_I_ to distinguish cancers. Previous studies have shown that using diffusional kurtosis imaging—a method similar to the one used herein but that can only assess the sum of the two kurtosis components MK_A_ and MK_I_—may provide increased value compared with standard DWI in PCa assessment.^38,39^ Another study did, however, not find reasons to replace DWI as the clinical standard to characterize PCa.^40^

Note that we report MD values higher than expected from previous studies.^41^ There are at least four different factors that could explain this: the data analysis model, the image resolution, the effective diffusion time, and the relaxation weighting. First, the data analysis was based on the so-called gamma model, which tends to overestimate the MD—in particular in the presence of high MK_I_.^42^ A separate analysis of the low b-value part of our data using a conventional monoexponential ADC analysis corroborates this, as it yielded values on average 25% lower than with the gamma model. In an analysis of prostate tissue, Hector et al similarly found that DKI analysis—comparable to the gamma model—yielded approximately 25% higher values of MD (referred to as ADC) than a monoexponential analysis of low b-value data.^43^ Second, the image resolution was low in this preliminary study (3 × 3 × 4 mm^3^). This limited the accuracy by which tumors can be delineated and increased the level of partial volume artifacts. As PC has low MD, partial inclusion of healthy tissue in the ROI increases the observed MD of the cancers. Third, the MD depends on the diffusion time of the gradient waveforms used for diffusion encoding. Shorter diffusion times are associated with higher MD values.^14^ The gradient waveforms used in b-tensor encoding have no well-defined diffusion time,^20^ however, as they use free gradient waveforms (Figure 1). Analysis of time-dependent diffusion can nevertheless be made using so-called spectral theory.^44^ The waveforms we use tend to oscillate as the q-vector builds the b-tensor (Figure 1),^19^ which means more energy at high frequencies. This corresponds to shorter diffusion times. How to efficiently design gradient waveforms to yield a given b-tensor while featuring specific diffusion times and high efficiency is still an open question,^20,45^ but will be essential to consider when planning future b-tensor encoding studies. Fourth—and last but certainly not least—we need to consider the TE. The prostate consists of both cellular and luminal tissues, which have different T_2_ relaxation times. At our TE of 101 ms, the luminal component is much less attenuated than the cellular component because of differences in T_2_ relaxation times (~300 ms vs 50 ms).^13^ As the T_2_ and the ADC of the luminal component are both higher than in the cellular component, the voxel-averaged ADC increases with TE.^46^ We can similarly expect a dependence of TE in MK_A_ and MK_I_, but this remains to be investigated. In this context we must also note that b-tensor encoding is less efficient than conventional encoding (lower b-values per unit time), which necessitates longer TEs. This leads to lower signal-to-noise ratio, which can be compensated by reducing the resolution at the expense of accurate tumor delineation. Longer echo times also affect the magnitude by which tissue components contribute to the signal. The impact of the tradeoffs between maximal b-value, resolution, and echo times in the context of b-tensor encoding in the prostate needs to be carefully analyzed, ideally using an MRI system with high-performance gradients which can acquire data at short echo times with high b-values.^47^

This study had four main limitations. First, it included only 17 subjects. This limits the statistical power for detection of differences between cancers of different Gleason scores. Future studies with larger sample sizes are needed to assess the diagnostic potential of b-tensor encoding in PCa. Second, the present protocol featured voxel sizes of 3 × 3 × 4 mm^3^, but higher resolution is warranted for more precise delineation of the lesions. A preliminary study suggests that 2 × 2 × 4 mm^3^ is achievable at a system with 80 mT/m gradients.^48^ Higher resolution gives less partial volume effects and thus reduce parameter bias. Third, the gradient waveforms applied here were not Maxwell compensated.^49^ This can lead to a negative bias in MK_I_ in locations far from the isocenter where the concomitant fields are larger. This probably had at most a mild impact on the present study because the prostate was positioned close to the isocenter. Fourth, zoomed-in sections of the histology sections shown in Figure 5 were obtained in regions manually selected to match the position of the MRI ROIs, so we lack voxel-to-voxel histology that is needed for careful assessment of what microstructure features that explain variations in diffusion MRI parameters.^50^ However, the histology results illustrated in Figure 5 provide an initial hypothesis about what causes variation in MK_A_ and MK_I_.

## 5 | CONCLUSION

We found that it was feasible to perform tensor-valued diffusion encoding in the prostate for the mapping of MK_A_ and MK_I_. We observed an elevated MK_I_ in PCa compared with normal tissues in the PZs and TZs. This suggests a potential of this parameter to support a PCa diagnosis; however, this hypothesis needs to be validated in a study with a larger sample size. Future studies would benefit from the use of an increased in-plane resolution for increased accuracy in the tumor delineation.

## Data Availability

The code used to analyze the data is available at https://github.com/markus-nilsson/md-dmri, SHA-1 hash 882b6ac.
